# Peroxisome proliferator-activated receptor-γ protects ERBB2-positive breast cancer cells from palmitate toxicity

**DOI:** 10.1186/bcr2240

**Published:** 2009-03-19

**Authors:** Antonis Kourtidis, Rekha Srinivasaiah, Richard D Carkner, M Julia Brosnan, Douglas S Conklin

**Affiliations:** 1 Department of Biomedical Sciences, Gen*NY*Sis Center for Excellence in Cancer Genomics, University at Albany, Discovery Drive, Rensselaer, NY 12144, USA; 2 Ordway Research Institute, Center for Cardiovascular Science, New Scotland Avenue, Albany, NY 12208, USA

## Abstract

**Introduction:**

Accumulation of fatty acids and neutral lipids in nonadipose tissues is cytotoxic. We recently showed that ERBB2-positive breast cancer cells produce significantly high amounts of fats, because of overexpression of the peroxisome proliferator-activated receptor (PPAR)γ-binding protein and the nuclear receptor NR1D1 (nuclear receptor subfamily 1, group D, member 1; Rev-erbα). These genes upregulate *de novo *fatty acid synthesis, which is a critical pathway for the energy production and survival of these cells. NR1D1 and PPARγ-binding protein are functionally related to PPARγ, a well established positive regulator of adipogenesis and lipid storage.

**Methods:**

The effects of GW9662 and exogenously added palmitate on breast cells (BT474, MDA-MB-361, MCF-7, and human mammary epithelial cells) in monolayer culture were assessed. Mass spectrometric quantitation of fatty acids and fluorescence-based high content microscopy assays of cell growth, apoptosis, triglyceride storage and reactive oxygen species production were used.

**Results:**

ERBB2-positive breast cancer cells are more sensitive to inhibition of PPARγ activity by the antagonist GW9662. PPARγ inhibition results in increased levels of total fats in the cells, mostly because of increased amounts of palmitic and stearic unsaturated acids. Administration of exogenous palmitate is lethal to ERBB2-positive but not to ERBB2-negative cells. GW9662 exacerbates the effects of palmitate addition on BT474 and MDA-MB-361 cells, but it has no significant effect on MCF-7 and human mammary epithelial cells. Palmitate administration results in a fivefold to tenfold greater increase in fat stores in ERBB2-negative cells compared with ERBB2-positive cells, which suggests that the ERBB2-positive cells have maximized their ability to store fats and that additional palmitate is toxic to these cells. Both PPARγ inhibition and palmitate administration result in increased reactive oxygen species production in BT474 cells. The cell death that results from this treatment can be counteracted by the antioxidant *N*-acetyl cysteine.

**Conclusions:**

Our findings indicate that PPARγ activity enables ERBB2-positive breast cancer cells, which produce high levels of fat, to convert fatty acids to triglycerides, allowing these cells to avert the cell death that results from lipotoxicity. Endogenous palmitate toxicity represents a genetically based property of ERBB2-positive breast cancer that can be exploited for therapeutic intervention.

## Introduction

Amplification of the *ERBB2 *oncogene is one of the most clinically relevant genetic changes in breast cancer and occurs in 10% to 34% of breast cancer cases. *ERBB2 *overexpression is a significant predictor of both overall survival and time to relapse [1]. The association of *ERBB2 *amplification with aggressive disease and poor clinical outcome in breast cancer has made ERBB2 an attractive therapeutic target. Trastuzumab, a human monoclonal antibody targeted against the extracellular domain of ERBB2, was widely hailed as the first next generation cancer therapy when it was introduced for the treatment of estrogen receptor-negative breast cancer. Its success has been modest. When used as single-agent therapy in patients with metastatic ERBB2-positive breast cancer, response rates ranging from 11% to 26% have been observed. Because a relatively large proportion of patients do not benefit from ERBB2-targeted therapy, it is likely that factors in addition to ERBB2 itself must influence the response of these tumors to this therapy. Recently, an RNA interference screen identified regulators of fat metabolism (including the peroxisome proliferator-activated receptor [PPAR]γ-binding protein [PBP] and the nuclear receptor NR1D1 [nuclear receptor subfamily 1, group D, member 1], a PPARγ target protein) as being relevant to the survival specifically of ERBB2-positive breast cancer cells, but not that of other breast cancer cells or normal mammary epithelial cells (Kourtidis A, Carkner RD, Eifert C, Brosnan MJ, Conklin DS; unpublished data). Both genes reside on the ERBB2 amplicon and are transcriptional regulators that positively affect expression of genes such as *FASN *(fatty acid synthase), *ACLY *(ATP citrate lyase) and *ACACA *(acetyl-coenzyme A carboxylase alpha), which are the three major enzymes of *de novo *fatty acid synthesis (Kourtidis A, Carkner RD, Eifert C, Brosnan MJ, Conklin DS; unpublished data). As a result, ERBB2-positive breast cancer cells contain significantly higher amounts of cellular fats, as compared with other breast cancer cell lines or normal cells, because of concomitant overexpression of *NR1D1 *and *PBP *genes (Kourtidis A, Carkner RD, Eifert C, Brosnan MJ, Conklin DS; unpublished data) [2].

The end product of *de novo *fatty acid synthesis, namely palmitate, and other saturated fatty acids like it, are toxic to cells. Palmitate has been shown to generate a variety of apoptotic signals [3,4]. In some cases these involve the synthesis of ceramide [5,6], whereas in others reactive oxygen species (ROS) are produced [3,7]. Studies conducted in a variety of cell types, including breast cancer cell lines [8,9], suggest that this lipotoxicity is specific for saturated fatty acids such as palmitate. Triglyceride accumulation in nonadipose cells represents a cellular defense mechanism against lipotoxicity [10]. Because exogenous unsaturated fatty acids have an impact on this process, it may represent a mechanism for effects of diet on cancer etiology.

Both PBP and NR1D1 are functionally related to PPARγ [11,12]. PPARγ expression is also higher in ERBB2-positive breast cancer cells [13]. These cells were more sensitive to inhibition of PPARγ with antagonists such as GW9662 and T0070907, as compared with other types of breast cancer cells or normal mammary epithelial cells (Kourtidis A, Carkner RD, Eifert C, Brosnan MJ, Conklin DS; unpublished data). PPARγ inhibition in ERBB2-positive breast cancer cells resulted in cell death and apoptosis, similar to the effects of PBP and NR1D1 inhibition (Kourtidis A, Carkner RD, Eifert C, Brosnan MJ, Conklin DS; unpublished data). PPARγ is a major regulator of adipogenesis and lipid homeostasis [14]. We sought to examine the reasons for the dependence of ERBB2-positive breast cancer cells on PPARγ for survival. In this context, we identified palmitate-induced lipotoxicity as a main effect of PPARγ inhibition in ERBB2-positive breast cancer cells.

## Materials and methods

### Cell culture and chemicals

Breast cancer cell lines BT474, MCF-7 and MDA-MB-361 were obtained from the American Type Culture Collection (Manassas, VA, USA). Human mammary epithelial cells (HMECs) were obtained from Cambrex (East Rutherford, NJ, USA). BT474 and MCF-7 cells were cultured in Dulbecco's modified Eagle's medium (Hyclone, Logan, UT, USA) supplemented with 10% fetal bovine serum (Hyclone) and 100 U/μl penicillin-streptomycin (Cellgro, Herndon, VA, USA); BT474 medium was also supplemented with ITS (insulin, transferring and selenium; Cellgro). MDA-MB-361 were cultured in RPMI-1640 (Hyclone) supplemented with 20% fetal bovine serum and 100 U/μl penicillin-streptomycin. HMECs were cultured in mammary epithelial growth medium (Cambrex). The PPARγ antagonist GW9662, the fatty acid palmitate, and the ceramide synthesis inhibitor fumonisin B1 were obtained from Sigma-Aldrich (St. Louis, MO, USA).

### Cell viability: proliferation assays

Cell viability after small hairpin RNA transfection or chemical treatments was assessed live cell counts after trypsinization and trypan blue staining using a hemocytometer. For high-throughput experiments, cells grown on 96-well plates were washed once with 1× phosphate-buffered saline (PBS), fixed with 2.5% formaldehyde, stained with Hoechst 33342 (Molecular Probes-Invitrogen, Carlsbad, CA, USA) and analyzed with an In Cell Analyzer 1000 (GE Healthcare, Piscataway, NJ, USA) high-content imaging system; cell counts and statistics were performed using In Cell Investgator 3.4 software (GE Healthcare).

### Reverse transcription polymerase chain reaction

Total RNA was extracted from cells using TRizol (Invitrogen, Carlsbad, CA, USA). cDNA was synthesized by reverse transcription of 2 μg of RNA in a 20 μl reaction using Moloney murine leukemia virus reverse transcriptase (Promega, Madison, WI, USA) at 42°C for 1 hour. PCR reactions were performed by using standard *Taq *polymerase (Fisher BioReagents, Fairlawn, NJ, USA) with the following primer pairs (forward and reverse, respectively): *PPARγ*, 5'-AGCCTCATGAAGAGCCTTCCA-3' and 5'-ACCCTTGCATCCTTCACAAGC-3'; fatty acid binding protein 4 (*FABP4*; *aP2*), 5'-GCATGGCCAAACCTAACATGAT-3' and 5'-CCTGGCCCAGTATGAAGGAAA-3'; hormone sensitive lipase (*HSL*), 5'-TACAAACGCAACGAGACAGGC-3' and 5'-TGTGATCCGCTCAAACTCAGC-3'; adipose tryglyceride lipase (*ATGL*), 5'-AGCTCATCCAGGCCAATGTCT-3' and 5'-GGTTGTCTGAAATGCCACCAT-3'; carnitine palmitoyltransferase 1 (*CPT-1*), 5'-TCACATTCAGGCAGCAAGAGC-3' and 5'-AATCGTGGATCCCAAAAGACG-3'; and glyceraldehyde-3-phosphate dehydrogenase (*GAPDH*), 5'-GCAAATTCCATGGCACCGT-3' and 5'-TCGCCCCACTTGATTTTGG-3'.

After the initial denaturation step (95°C for 3 minutes), PCR reactions consisted of 30 to 35 cycles of a 95°C step (15 seconds), a 52 to 55°C step (15 seconds), and a 72°C step (20 seconds), followed by a final elongation step at 72°C (5 minutes). PCR products were separated on 2% agarose-ethidium bromide gels. For quantitative determination of PCR product, a real-time reverse transcription PCR (RT-PCR) was performed on an ABI PRISM 7900 HT Sequence Detection System (Applied Biosystems, Foster City, CA, USA), using SYBR Green PCR Master Mix (Applied Biosystems). Primer pairs were the same as those used in regular RT-PCR. PCR reactions consisted of an initial incubation at 95°C (2.5 minutes) and 40 cycles of a 95°C step (15 seconds) and a 60°C step (60 seconds). Product levels were calculated after normalization with GAPDH or β-actin controls.

### Immunofluoresence

Immunofluoresence was performed on cells grown and treated either in 96-well plates or on cover slips in 24-well plates. In all cases, cells were fixed after treatment with 2.5% formaldehyde, washed with 1× PBS, permeabilized with 0.1% Triton-X 100 (Fisher Chemicals, Fairlawn, NJ, USA), blocked with 3% normal goat serum (Sigma-Aldrich), incubated with a 1:50 to 1:200 dilution of the primary antibody, washed with 1× PBS, incubated with a 1:800 dilution of the secondary antibody, washed again with 1× PBS, and finally stained with Hoechst 33342 (Molecular Probes-Invitrogen). Cells stained on 96-well plates were imaged using the In Cell Analyzer 1000 (GE Healthcare) and signal measurements and statistics were performed by the In Cell Investigator 3.4 software (GE Healthcare). Cells immunostained on cover slips were imaged by using a Leica TCS SP5 confocal microscope system (Leica Microsystems Inc., Bannockburn, IL, USA). Antibodies used were anti-activated Bax (6A7; BD Pharmingen, San Jose, CA, USA) and Alexa Fluor 568 goat anti-rabbit IgG (#A-11011; Invitrogen).

### Metabolic assays

For detection of neutral fat stores, cells were grown on 96-well plates, fixed with 2.5% formaldehyde, washed with 1× PBS, stained with 10 μg/ml 4,4-difluoro-1,3,5,7,8-pentamethyl-4-bora-3a,4a-diaza-*s*-indacene (BODIPY 493/503; Molecular Probes), and counter-stained with Hoechst 33342 (Molecular Probes) for nuclei identification. Cells were imaged using the In Cell Analyzer 1000 and pictures were analyzed using the In Cell Investigator 3.4 software.

For fatty acid detection and quantification, total cellular lipids were extracted in accordance with a procedure described previously [15]. Briefly, approximately 10^7 ^cells were pelleted and re-suspended in 3 ml chloroform:methanol (1:2) weight/0.05% butylated hydroxytoluene (BHT) to extract lipids. To monitor the recovery of fatty acids, 100 μg of heptadecanoic acid was added to each sample, before lipid extraction. Samples were centrifuged to remove cellular debris and chloroform and distilled water was added to form a biphasic solution. The two phases were separated by centrifugation and organic phases were transferred to a new tube and dried under nitrogen gas. Fats were then re-suspended in 250 μl toluene + 500 μl 1% sulfuric acid in methanol and incubated at 50°C overnight under nitrogen gas, followed by two steps of 1.25 ml 5% NaCl – 1.25 ml hexane extraction. Hexane extractions were combined, washed with 1.0 ml 2% NaHCO_3_, run through Na_2_SO_4 _columns, and dried under nitrogen gas. The fatty acid methyl esters were re-suspended in 1.0 ml methyl acetate and were analyzed by gas chromatography/mass spectrometry using an Agilent 6890 series gas chromatograph (Agilent Technologies Inc., Santa Clara, CA, USA) equipped with a 5873 mass-selective detector. Part of the extraction was used for quantification of total and individual fatty acids under mass spectrometry and part for thin layer chromatography separation of triglycerides by a toluene-based system.

### Reactive oxygen species assay

O_2_^- ^generation was measured by using hydroethidine (Molecular Probes-Invitrogen, Carlsbad, CA, USA). Cells were incubated with a final concentration of 10 μmol/l of the dye for 30 minutes, and then fixed with 2.5% formaldehyde, stained with Hoechst 33342 and imaged with an In Cell Analyzer 1000. The hydroethidine signal was quantified using the In Cell Investigator 3.4 software.

### Statistical analysis

The Student's two-tailed *t*-test was employed for the calculation of *P *values.

## Results

### PPARγ inhibition results in increased levels of fats

**Figure 1:**
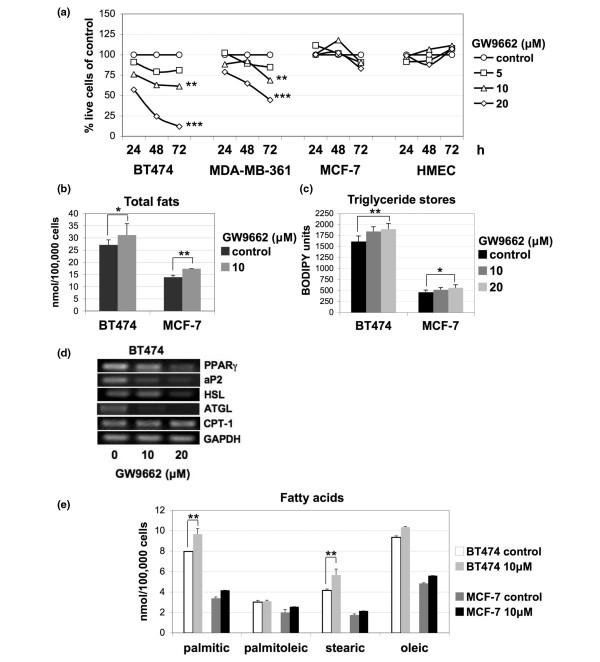
PPARγ inhibition results in ERBB2-positive cancer cell death and increased levels of fats. **(a) **Cell counts of BT474, MDA-MB-361, MCF-7, and human mammary epithelial cell (HMEC) cells treated with vehicle, 5, 10, and 20 μmol/l of the peroxisome proliferators-activated receptor (PPAR)γ antagonist GW9662 for 24, 48, and 72 hours. Results are presented as percentage of control (vehicle). Error bars indicate the standard deviation from three individual experiments. ***P < 0.003, **P < 0.05. **(b) **Quantification (nmol/100,000 cells) of total cellular fats from BT474 and MCF-7 whole cell extracts by mass spectrometry after treatment with vehicle or 10 μmol/l of GW9662. Error bars indicate the standard deviation from two experiments. **P = 0.01, *P = 0.3). **(c) **Quantification of neutral fat stores of BT474 and MCF-7 cells treated with vehicle or 10 μmol/l of GW9662 by using the BODIPY 493/503 lipid probe staining. Error bars indicate the standard deviation from three individual experiments. **P = 0.05, *P = 0.1. **(d) **RT-PCR of BT474 cells treated with vehicle, 10, or 20 μmol/l of GW9662. Glyceraldehyde-3-phosphate dehydrogenase (GAPDH) was used as control. **(e) **Quantification (in nmol/100,000 cells) by mass spectrometry of palmitic, palmitoleic, stearic, and oleic fatty acids from BT474 and MCF-7 whole cell extracts after treatment with vehicle or 10 μmol/l of GW9662. Error bars indicate the standard deviation from two experiments. **P < 0.05. aP2, fatty acid binding protein 4 (FABP4); ATGL, adipose tryglyceride lipase; CPT, carnitine palmitoyltransferase; HSL, hormone sensitive lipase.

Inhibition of PPARγ transcriptional activity using the antagonist GW9662 resulted in increased cell death, specifically of the ERBB2-positive BT474 and MDA-MB-361 breast cancer cells, in a time-dependent and dosage-dependent manner, but not of the ERBB2-negative MCF-7 or normal HMECs (Figure 1a). PPARγ is a major adipogenesis regulator, and therefore we examined cells for changes in fat content. Because we were primarily interested in learning why the ERBB2-positive cells were sensitive to treatment with PPARγ antagonists whereas the EBRB2-negative cells were relatively resistant, we initially focused on differences between an ERBB2-positive (BT474) and an ERBB2-negative cell line (MCF-7). Overall, BT474 cells contain twice the amount of total fats (Figure 1b) and three times more stored triglycerides compared with MCF-7 cells (Figure 1c). This is consistent with our previous studies showing overexpression of NR1D1, PBP, and FASN, causing higher fat content in ERBB2-positive cells (Kourtidis A, Carkner RD, Eifert C, Brosnan MJ, Conklin DS; unpublished data). In order to quantify the total amount of fats after PPARγ inhibition, we used sublethal concentrations of GW9662, because at higher concentrations extensive cell death occurs (Figure 1a) preventing reliable measurement. Mass spectrometry revealed a minor increase in the total amount of fats in both cell lines (Figure 1b). A similar increase was observed in triglyceride stores (Figure 1c). These findings were somewhat surprising, because it was expected that inhibition of PPARγ would lead to decreased fat levels.

To investigate the cause of the nearly steady levels of stored fats, the message levels of PPARγ target genes related to triglyceride degradation were examined. The major target of PPARγ, namely fatty acid binding protein *aP2 *[16], was downregulated upon PPARγ inhibition, indicating that GW9662 effectively blocks PPARγ transcriptional activity (Figure 1d). Interestingly, *PPARγ *mRNA itself was downregulated by the GW9662 antagonist (Figure 1d). The two major lipases involved in the breakdown of triglycerides, *HSL *and *ATGL*, were downregulated upon use of GW9662 (Figure 1d), as expected [17,18]. It has been shown that HSL, in order to effectively continue triglyceride degradation, interacts with aP2, which binds and removes fatty acids from lipid droplets [19]. Therefore, the increase in triglyceride levels after downregulation of *aP2 *and *HSL*, as well as *ATGL*, is consistent with attenuation of lipolysis. Furthermore, mRNA levels of carnitine palmitoyltransferase 1, the key enzyme in fatty acid β-oxidation, were not affected by GW9662 treatment, showing that fatty acid degradation was not induced after PPARγ inhibition (Figure 1d). Taken together, these findings provide clues for the observed lack of triglyceride degradation after PPARγ inhibition.

In order to examine levels of individual fatty acids, we examined the fat content of the BT474 and MCF-7 cells by mass spectrometry. The results showed that that the higher fat levels after PPARγ inhibition at sublethal concentrations of GW9662 (10 μmol/l) were primarily due to increased amounts of the saturated palmitic and stearic fatty acids (increases of 1.7 and 1.6 nmol/100,00 cells, respectively; *P *< 0.05; Figure 1e). These two fatty acids, together with the unsaturated palmitoleic and oleic acids, comprised almost 80% of the total amount of fats in the cells examined, with palmitate content reaching about 30% (Figure 1b,e). The increase in palmitic and stearic acids was also higher by 0.9 and 1.2 nmol/100,000 cells, respectively, in BT474 cells compared with MCF-7 cells (Figure 1e). Therefore, inhibition of PPARγ leads to increased levels of fats, mainly due to the increased production of palmitate.

### Exogenous administration of palmitate is toxic specifically for ERBB2-positive cells

**Figure 2:**
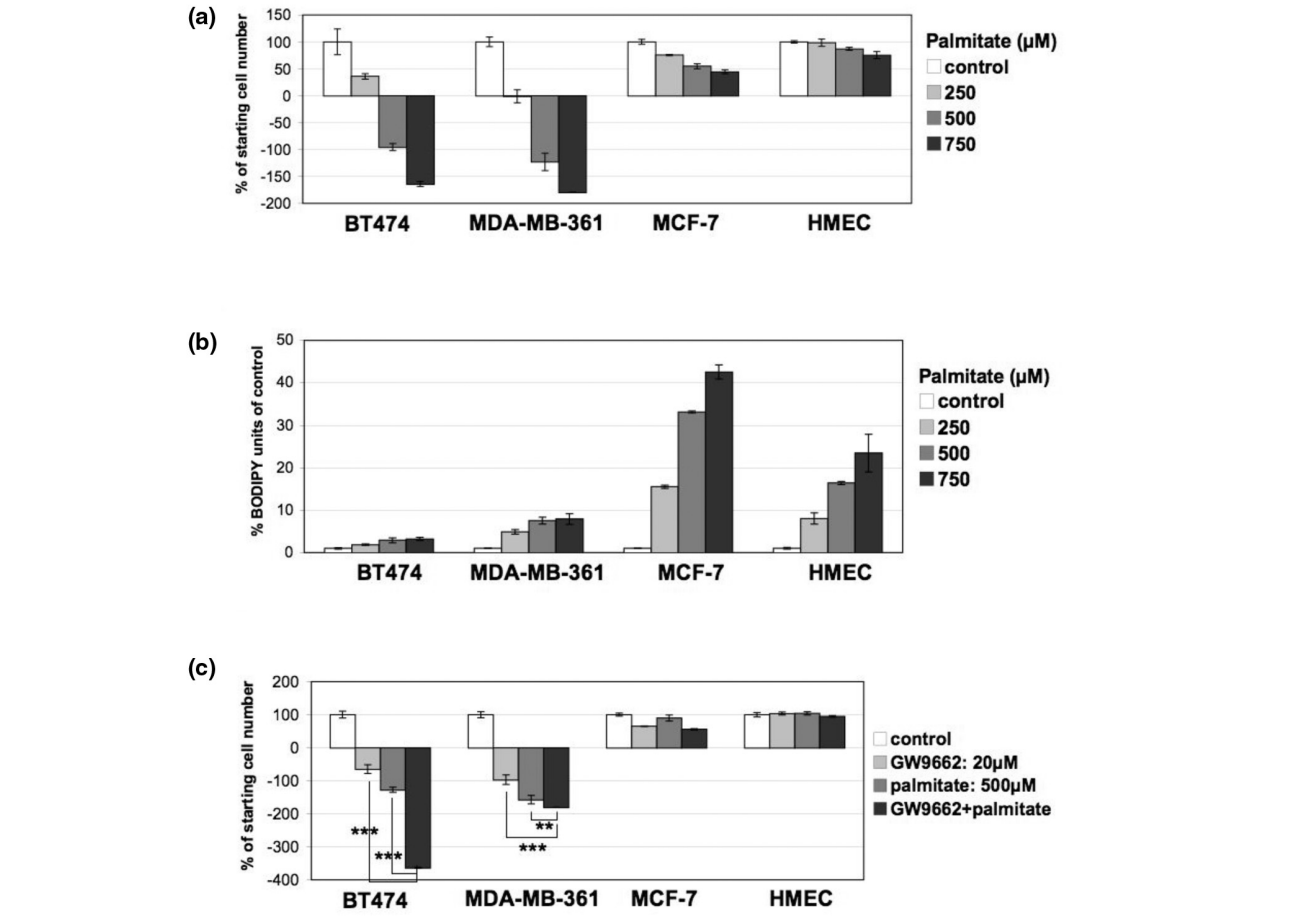
Administration of exogenous palmitate is toxic specifically to ERBB2-positive cells. **(a) **BT474, MDA-MB-361, MCF-7 and human mammary epithelial cell (HMEC) cells treated with 250, 500, and 750 μmol/l palmitate for 72 hours. Cell counts are shown as percentage of seeded cell number at 0 hours. Error bars indicate the standard deviation from three individual experiments). **(b) **Quantification of neutral fat stores of BT474, MDA-MB-361, MCF-7, and HMEC cells treated with 250, 500, and 750 μmol/l palmitate for 72 hours by using the BODIPY 493/503 lipid probe staining. Error bars indicate the standard deviation from three individual experiments. **(c) **BT474, MDA-MB-361, MCF-7, and HMEC cells treated with 500 μmol/l palmitate and/or 20 μmol/l GW9662 for 72 hours. Cell counts are shown as percentage of seeded cell number at 0 hours. Error bars indicate the standard deviation from three individual experiments. ***P < 0.0005, **P = 0.03.

The end product of *de novo *fatty acid synthesis, palmitate, is toxic to cells. The relatively high levels of activity of the fatty acid synthesis pathway in the ERBB2-positive breast cancer cells are likely to require a mechanism for overcoming the toxic effects of palmitate excess. PPARγ inhibition correlated with increased palmitate content and cell death in BT474 cells. Therefore, PPARγ activity in the highly fat-producing ERBB2-positive cells may serve in the detoxification of cells from palmitate excess, by affecting its storage as triglycerides. To investigate whether excess palmitate could be the trigger of cell death, cells were grown with 250, 500, and 750 μmol/l of palmitate. BT474 and MDA-MB-361 cells were highly sensitive to exogenously added palmitate, which caused cell death in a dose-dependent manner, resulting after 72 hours in 1.5 to 1.8 times less cells than the initial population at 750 μmol/l (Figure 2a). In contrast, palmitate only decreased proliferation of MCF-7 cells and HMECs by 50% and 25%, respectively, at the highest concentrations, without causing cell death (Figure 2b). Interestingly, whereas BT474 cells appear to have accumulated about two to three times and MDA-MB-361 cells about seven to eight times more triglycerides after treatment with 500 to 750 μmol/l palmitate, MCF-7 cells were able to increase their fat stores by 30 to 40 times and HMECs by 16 to 25 times under the same conditions, without any effect in their viability (Figure 2b). The effect of palmitate treatment on ERBB2-positive cells was exacerbated by simultaneous addition of GW9662, whereas the same treatments were without effect on MCF-7 cell and HMEC viability (Figure 2c).

**Figure 3:**
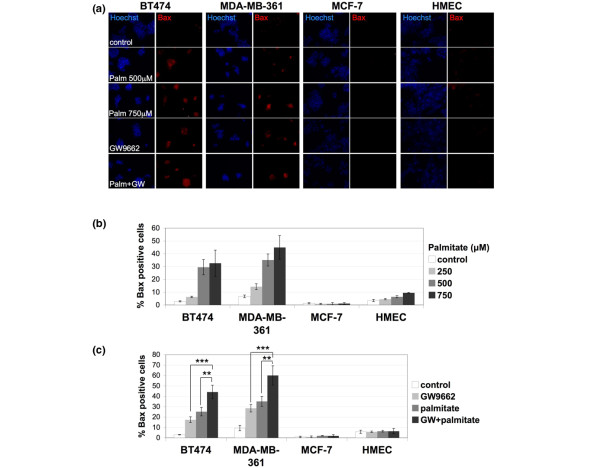
Palmitate toxicity of ERBB2-positive cells is due to increased apoptosis. **(a) **Images of BT474, MDA-MB-361, MCF-7, and human mammary epithelial cell (HMEC) cells stained for activated Bax signal that were treated with 500 or 750 μmol/l palmitate, 20 μmol/l GW9662, or 500 μmol/l palmitate plus 20 μmol/l GW9662, all for 72 hours. Hoechst 33243 was used for nuclear staining. **(b, c) **Quantification of activated Bax signal. Error bars indicate the standard deviation from three individual experiments. ***P < 0.004, **P = 0.01.

Further examination of the cause of BT474 cell death after palmitate addition showed that this is due to increased apoptosis. Palmitate administration resulted in increased activated Bax signal, in a dose-dependent manner (Figure 3a,b), which was also further induced by GW9662 addition (Figure 3a,c). Neither palmitate nor GW9662 addition resulted in Bax activation in MCF-7 cells or HMECs. The above findings support the idea that ERBB2-positive cells have near toxic levels of endogenously produced palmitate, which lead to apoptosis when PPARγ activity is inhibited.

### Peroxisome proliferator-activated receptor-γ inhibition and palmitate administration in ERBB2-positive cells result in reactive oxygen species production

**Figure 4:**
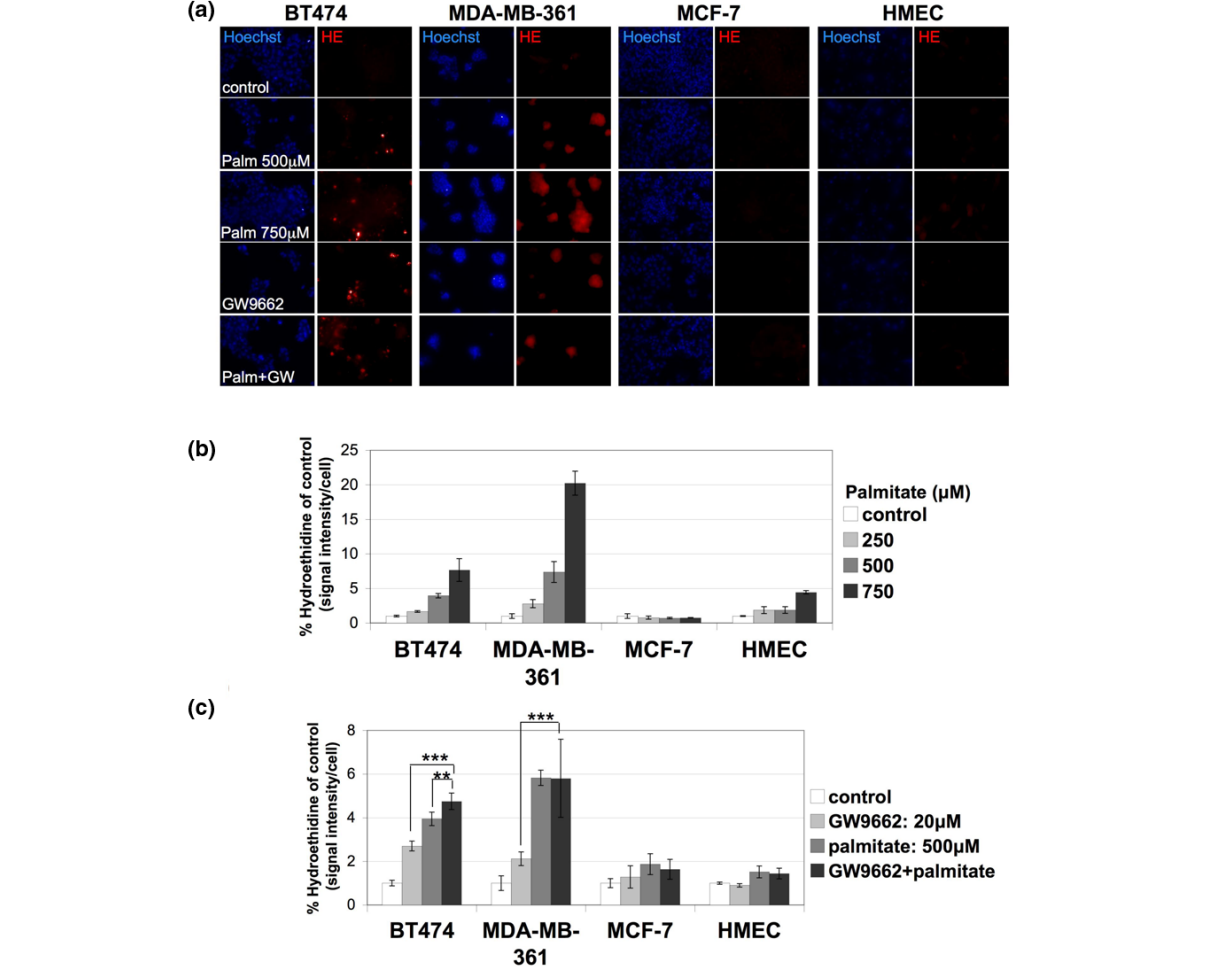
PPARγ inhibition and palmitate treatment result in increased ROS. **(a) **BT474, MDA-MB-361, MCF-7, and human mammary epithelial cell (HMEC) cells were treated with 500 or 750 μmol/l palmitate, 20 μmol/l GW9662, or 500 μmol/l palmitate plus 20 μmol/l GW9662 for 72 hours and stained with hydroethidine (HE) for detection of O_2_^- ^species. Hoechst 33243 was used for nuclear staining. **(b) **Quantification of HE signal of BT474, MDA-MB-361, MCF-7, and HMEC cells treated with 250, 500 and 750 μmol/l palmitate for 72 hours. Error bars indicate the standard deviation from three individual experiments. **(c) **Quantification of HE signal of BT474, MDA-MB-361, MCF-7, and HMEC cells treated with 500 μmol/l palmitate and/or 20 μmol/l GW9662 for 72 hours. Error bars indicate the standard deviation from three individual experiments. ***P < 0.02, **P = 0.05. PPAR, peroxisome proliferator-activated receptor; ROS, reactive oxygen species.

It is well established that palmitate toxicity can be mediated by ROS production. In studies conducted in Chinese hamster ovary cells, intracellular palmitate accumulation induced apoptosis through generation of ROS [7]. To test whether PPARγ blockade by GW9662 and palmitate toxicity in ERBB2-positive cells is due to ROS production, we used hydroethidine, an O_2_^- ^species fluorescent indicator, to monitor cells after these treatments. Palmitate treatment induced a fourfold to sevenfold increase in hydroethidine signal in BT474 cells and a sevenfold to 20-fold increase in MDA-MB-361 cells, when administering 500 to 750 μmol/l of the fatty acid (Figure 4a,b). Palmitate treatment did not result in increased hydroethidine signal in MCF-7 cells at any concentration and induced only a moderate increase in HMECs at the highest concentration (Figure 4a,b). This increase in HMECs does not correlate with cell death (Figure 2a) and produces only a modest increase in activated Bax signal when compared with the activated Bax increase observed in the BT474 and MDA-MB-361 cells under these conditions (Figure 3b). Therefore, the moderate increase of the hydroethidine signal in HMECs treated with 750 μmol/l palmitate is not suggestive of palmitate sensitivity in these cells. GW9662 also resulted in an almost threefold increase in hydroethidine signal in BT474 and twofold in MDA-MB-361 cells, but not in MCF-7 cells or HMECs (Figure 4a,c). Induction of O_2_^- ^species in BT474 cells by palmitate was exacerbated by GW9662 addition (Figure 4a,c), consistent with the similar exacerbation of apoptosis under the same conditions (Figure 3c).

**Figure 5:**
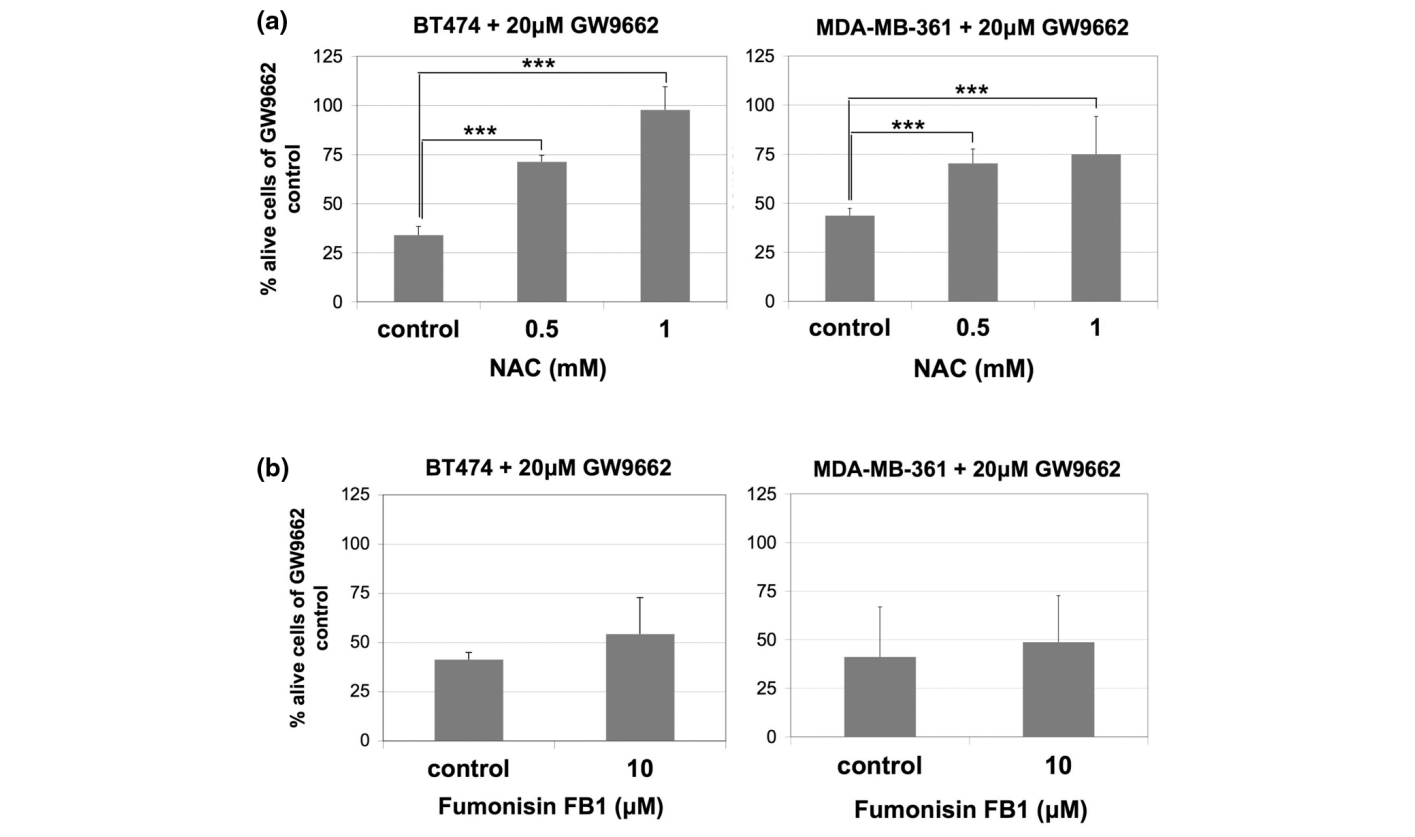
ERBB2-positive cells' sensitivity to palmitate is through ROS formation and ceramide production. **(a) **BT474 and MDA-MB-361 cells were treated with vehicle or 20 μmol/l GW9662 together with 0, 0.5, and 1 μmol/l of *N*-acetyl-cysteine (NAC) for 72 hours. Live cells were counted and presented as percentage of the GW9662 control. Error bars indicate the standard deviation from three individual experiments. ***P < 0.05. **(b) **BT474 and MDA-MB-361 cells were treated with vehicle or 20 μmol/l GW9662 together with 0 and 10 μmol/l of the ceramide synthesis inhibitor fumonisin B1 for 72 hours. Live cells were counted and presented as percentage of the GW9662 control. Error bars indicate the standard deviation from three individual experiments. ROS, reactive oxygen species.

To determine whether ROS is the reason for cell death, BT474 and MDA-MB-361 cells were treated with GW9662 together with the antioxidant *N*-acetyl-cysteine. *N*-acetyl-cysteine rescued cells from PPARγ inhibition, indicating that oxidative damage caused by increased ROS is the reason for cell death (Figure 5a). Another mediator of palmitate toxicity is ceramide synthesis. To examine whether PPARγ inhibition results in ceramide-mediated toxicity, BT474 and MDA-MB-361 cells treated with GW9662 were simultaneously treated with the ceramide synthesis inhibitor fumonisin B1. Fumonisin B1 only moderately rescued BT474 cells from PPARγ inhibition-mediated cell death (Figure 5b), indicating that palmitate toxicity caused by PPARγ inhibition in BT474 cells is largely ceramide indepedent. Therefore, PPARγ inhibition results in ROS production that causes apoptosis in BT474 cells.

## Discussion

ERBB2-positive cells possess significantly higher amounts of tryglyceride stores than do other cell types (Kourtidis A, Carkner RD, Eifert C, Brosnan MJ, Conklin DS; unpublished data; Figure 1b–e). It has been shown that PBP and NR1D1 promote adipogenesis, and therefore fat storage, because of their functional relationship with PPARγ (Kourtidis A, Carkner RD, Eifert C, Brosnan MJ, Conklin DS; unpublished data) [11,12]. PPARγ activity is necessary for the viability specifically of ERBB2-positive breast cancer cells, in a similar manner to PBP and NR1D1 (Kourtidis A, unpublished data; Carkner RD, Eifert C, Brosnan MJ, Conklin DS; Figure 1a). Our findings indicate that apoptosis due to PPARγ inhibition is consistent with endogenous palmitate toxicity. PPARγ enables ERBB2-positive breast cancer cells to convert fatty acids to triglycerides in order to avert lipotoxicity caused by the significantly high levels of fats that these cells produce. The decreased ability of BT474 and MDA-MB-361 cells to accumulate more fats after exogenous supplementation of palmitate (Figure 2b,d) is in agreement with the idea that these cells have near toxic levels of endogenously produced palmitate. Exogenous palmitate that has no effects on MCF-7 cell or HMEC viability is lethal to the ERBB2-positive cells. These results underscore the importance of triglyceride accumulation as a cellular defense against lipotoxicity [10] in cells that have abnormally high levels of fatty acid synthesis activity.

It has been established that cancer cells depend on an altered cellular physiology. As an example, cancer cells favor aerobic glycolysis instead of oxidative phosphorylation for energy production, a phenomenon described by Warburg several decades ago [20]. Fatty acid synthesis has been proposed to facilitate this mode of energy production in several cancer cells [21,22]. Over-expression of *PBP *and *NR1D1 *in ERBB2-positive breast cancer cells causes these cells to store fatty acids at 10 times the level of other breast cancer cells (Kourtidis A, Carkner RD, Eifert C, Brosnan MJ, Conklin DS; unpublished data) [2]. These two genes act coordinately to upregulate *de novo *fatty acid synthesis, enabling ERBB2-positive cells to regenerate their NAD^+ ^by consuming nicotinamide adenine dinucleotide phosphate (NADPH) that is necessary for fatty acid synthesis. As a result, the cells can continue to catabolize glucose and maintain their metabolic balance (Kourtidis A, Carkner RD, Eifert C, Brosnan MJ, Conklin DS; unpublished data). ERBB2 has been also shown to have a positive bidirectional relationship with FASN, in this way influencing *de novo *fatty acid synthesis [23]. The tight genetic linkage between *PBP*, *NR1D1*, and *ERBB2 *on the 17q12-21 amplicon commonly found in breast cancers [24] suggests that ERBB2-positive breast cancer cells are genetically preprogrammed to depend on fatty acid synthesis for energy production.

The generation of ROS and the biosynthesis of ceramide have been proposed as possible mechanisms of lipotoxicity [4]. Increased ROS production in BT474 cells following PPARγ inhibition, along with cell rescue after treatment with antioxidants, support the notion that ROS production is the mediator of palmitate toxicity in BT474 cells. However, inhibition of ceramide synthesis under the same conditions resulted in only a 27% increase in BT474 cell viability, showing that palmitate toxicity is mainly independent of ceramide production.

Several studies have shown that supplementation of saturated rather than unsaturated fatty acids is toxic to cells [25]. When ERBB2-negative breast cancer cells like MDA-MB-231 were involved, only palmitate, but not oleate, was shown to induce apoptosis [9]. It has been proposed that this is because unsaturated fats are more efficiently metabolized into triglycerides [26] and that they can rescue cells from lipotoxicity by channeling saturated fats like palmitate to triglyceride stores. Nevertheless, a number of reports have indicated that the Mediterranean diet, which is rich in the unsaturated oleate-containing olive oil, has anti-oncogenic properties against ERBB2-positive breast cancer [27,28]. Similar effects were seen for other unsaturated fatty acids, like the omega-3 polyunsaturated docosahexaenoic acid [29] and the omega-6 polyunsaturated γ-linolenic acid [30]. PPARγ inhibition in BT474 cells resulted in higher levels mainly of saturated rather than unsaturated fats (Figure 1d,e), and we confirmed that the saturated fatty acid palmitate is specifically toxic to these cells. However, the unsaturated oleate was also increased by PPARγ inhibition (Figure 1e), and we observed that oleate treatment produced similar toxic effects on BT474 cells (data not shown). This indicates that it is not the type of fatty acid that is responsible for the toxicity in these cells, but rather the overall high levels of fats caused by the significantly upregulated *de novo *fatty acid synthesis. ERBB2-positive cells have maximized their ability to store fats, and therefore additional supplementation of fatty acids, or interruption of their ability to store them by PPARγ inhibition, is lethal to these cells.

Excess of palmitate in ERBB2-positive cells could also feedback to inhibit fatty acid synthesis. It has been shown that high-fat diet downregulates both FASN and malic enzyme 1 [31]. Both enzymes are necessary for *de novo *fatty acid synthesis, because FASN is the enzyme that catalyzes palmitate synthesis from acyl-coenzyme A and malonyl-coenzyme A using NADPH, whereas malic enzyme 1 supplies FASN with NADPH. ERBB2-positive breast cancer cells are sensitive to inhibition of both FASN and malic enzyme 1 genes (Kourtidis A, Carkner RD, Eifert C, Brosnan MJ, Conklin DS; unpublished data). In this case, PPARγ activity is not only important for detoxifiying the cells but also for securing palmitate into fat stores, in order for FASN and malic enzyme 1 to continue to function. Further examination is needed to confirm this hypothesis and establish this feedback loop, which could provide several missing links in the regulation of this metabolic process and, therefore, further ways to eradicate this particularly aggressive form of cancer.

## Conclusions

The genetic linkage between *NR1D1*, *PBP*, and *ERBB2 *causes co-overexpression of these gene products such that ERBB2-positive breast cancer cells are preprogrammed to depend on fatty acid synthesis for energy production and survival. This study shows that the adipogenic transcription factor PPARγ is critical for ERBB2-positive breast cancer cells to convert the high levels of fatty acids that they produce into triglycerides, allowing them to avert the cell death that results from lipotoxicity. The decreased ability of ERBB2-positive cells to accumulate fats after exogenous supplementation of palmitate supports the notion that these cells have near toxic levels of endogenously produced palmitate. Increasing palmitate levels by inhibiting PPARγ or through the direct administration of palmitate results in cell death. Endogenous palmitate toxicity represents a genetically based property of ERBB2-positive breast cancer that can be exploited for therapeutic intervention.

## Abbreviations

ATGL: adipose tryglyceride lipase; FASN: fatty acid synthase; aP2: fatty acid binding protein 4 (FABP4); GAPDH: glyceraldehyde-3-phosphate dehydrogenase; HMEC: human mammary epithelial cell; HSL: hormone sensitive lipase; NADPH: nicotinamide adenine dinucleotide phosphate; NR1D1: nuclear receptor subfamily 1, group D, member 1; PBP: peroxisome proliferator-activated receptor-γ-binding protein; PBS: phosphate-buffered saline; PPAR: peroxisome proliferator-activated receptor; ROS: reactive oxygen species; RT-PCR: reverse transcription polymerase chain reaction.

## Competing interests

The authors declare that they have no competing interests.

## Authors' contributions

AK carried out all cell-based studies, participated in fatty acid analysis studies, and drafted the manuscript. RS participated in the ROS studies. MJB and RC carried out fatty acid analysis. DC conceived of the study, participated in its design and coordination, and helped to draft the manuscript. All authors read and approved the final manuscript.
